# A Major Locus for Chloride Accumulation on Chromosome 5A in Bread Wheat

**DOI:** 10.1371/journal.pone.0098845

**Published:** 2014-06-03

**Authors:** Yusuf Genc, Julian Taylor, Jay Rongala, Klaus Oldach

**Affiliations:** 1 School of Agriculture, Food and Wine, University of Adelaide, Waite Campus, Glen Osmond, South Australia, Australia; 2 South Australian Research and Development Institute, Plant Genomics Centre, Waite Campus, Glen Osmond, South Australia, Australia; Institute of Botany, Chinese Academy of Sciences, China

## Abstract

Chloride (Cl^−^) is an essential micronutrient for plant growth, but can be toxic at high concentrations resulting in reduced growth and yield. Although saline soils are generally dominated by both sodium (Na^+^) and Cl^−^ ions, compared to Na^+^ toxicity, very little is known about physiological and genetic control mechanisms of tolerance to Cl^−^ toxicity. In hydroponics and field studies, a bread wheat mapping population was tested to examine the relationships between physiological traits [Na^+^, potassium (K^+^) and Cl^−^ concentration] involved in salinity tolerance (ST) and seedling growth or grain yield, and to elucidate the genetic control mechanism of plant Cl^−^ accumulation using a quantitative trait loci (QTL) analysis approach. Plant Na^+^ or Cl^−^ concentration were moderately correlated (genetically) with seedling biomass in hydroponics, but showed no correlations with grain yield in the field, indicating little value in selecting for ion concentration to improve ST. In accordance with phenotypic responses, QTL controlling Cl^−^ accumulation differed entirely between hydroponics and field locations, and few were detected in two or more environments, demonstrating substantial QTL-by-environment interactions. The presence of several QTL for Cl^−^ concentration indicated that uptake and accumulation was a polygenic trait. A major Cl^−^ concentration QTL (5A; *barc56/gwm186*) was identified in three field environments, and accounted for 27–32% of the total genetic variance. Alignment between the 5A QTL interval and its corresponding physical genome regions in wheat and other grasses has enabled the search for candidate genes involved in Cl^−^ transport, which is discussed.

## Introduction

Worldwide, salinity poses a serious threat to agricultural production, with globally salt-affected soils (including saline and sodic soils) totalling around 830 million hectares [1]. Saline soils are generally dominated by sodium and chloride ions, and the ability of crop plants to exclude these ions is often equated to salinity tolerance-ST (i.e. improved growth or yield under salinity stress). Despite intensive research and numerous scientific reports over several decades, there is a lack of consensus on whether Na^+^ exclusion is always a useful trait to select for to improve ST, at least in the case of bread wheat. Few studies have shown good phenotypic correlation between Na^+^ exclusion and ST [2]–[5], while others have reported weak [6], [7] or no correlation [8]–[10]. In contrast to Na^+^ exclusion, there has been very little research on the role of Cl^−^ exclusion and its contribution to ST [3], [11]–[13]. Similar to Na^+^ exclusion, these few studies on Cl^−^ reported either significant phenotypic correlation or no correlation with ST. It is clear that future research needs to place equal emphasis on the impact of Cl^−^ in ST as not only Na^+^ but also Cl^−^ is present at toxic concentrations in saline-affected growth media used in the assessment of ST [13], [14].

In screening studies for ST, sodium chloride is the most commonly used salt. Despite both Na^+^ and Cl^−^ ions being present at toxic concentrations in growth media, there has been a lack of interest in Cl^−^, which may be attributed to the earlier reports that Na^+^ was more toxic than Cl^−^ [15]. Although these authors cautioned about assumptions made from extrapolations of two lines of bread wheat, their findings were not verified with a large set of genotypes differing in ST. This was later questioned by Martin and Koebner [16] on the basis that while attempting to separate the toxic effects of Na^+^ and Cl^−^, the authors used phytotoxic concentrations of nitrate in their experiments. Martin and Koebner [16] concluded that Cl^−^ was more toxic than Na^+^, but the full toxic effect was apparent only when Na^+^ and Cl^−^ were present simultaneously. However, more recent studies in barley found that Na^+^ and Cl^−^ had a similar effect upon plant growth [17]. While the issue of whether Na^+^ or Cl^−^ is more detrimental to plant growth remains controversial, it is appropriate to measure the concentration of both ions in the plant and to determine their relevance to ST for reasons mentioned earlier. Munns and Tester [18] argued that toxicity of Na^+^ versus Cl^−^ can be best studied through genetic approaches as the alternative method of using different salts has to date produced equivocal results. The potential of the genetic approach has not been employed extensively.

Chloride is an essential micronutrient and has several functions in plant metabolism; enzyme activation, photosynthesis, a counter ion for cation transport, osmoregulation, and movement of stomata. Like most anions, it is weakly bound to soil particles, mostly in a soluble form in soil solution, and at high concentrations can be toxic to plant growth. Critical toxicity concentration in plants was estimated at 110–200 mmol kg^−1^ DW (4,000–7,000 mg kg^−1^ DW) and 420–1400 mmol kg^−1^ DW (15,000–50,000 mg kg^−1^ DW) for sensitive and tolerant species, respectively [19]. At present there is very little information on genetic control mechanisms of Cl^−^ exclusion in plant species. A better understanding of genetic control of Cl^−^ exclusion and identification of molecular markers has the potential to speed up breeding for a complex trait such as ST. To date, rice and barley appear to be the only cereal species in which genetic control of Cl^−^ exclusion has been investigated. In two studies involving rice F_2_ and RIL populations derived from a cross between salt tolerant and salt-sensitive varieties CSR27 and MI48 respectively, few Cl^−^ concentration QTL were co-located with Na^+^ concentration QTL, while others mapped to different regions [20], [21]. Similar findings were also reported in barley [22], [23]. If QTL for Na^+^ and Cl^−^ concentration map to different locations, this would suggest separate genetic control mechanisms for regulation of these ions. At present, we are not aware of any reports on QTL mapping of Cl^−^ concentration in wheat.

Screening for ST is usually conducted in one of three main environments: hydroponics, soil-based pot assays or field trials. Due to inherent difficulties with field screening such as non-uniform distribution of salinity throughout the experimental area, fluctuations in rainfall and potential nutritional deficiencies, controlled environments are often preferred. However, as reported in recent studies [24], the results of controlled environments can be quite different from those of field environments and, therefore, require verification. The only two genetic studies on Cl^−^ accumulation to date have been conducted in hydroponics, and how these results correlate to field environments is unknown. It is important to note that the ability of controlled environmental studies to predict yield responses in the field is rarely addressed in the scientific literature, and the validation of controlled environmental studies in the field is important for plant breeding.

In previous studies in hydroponics and field trials [24], [25], we reported QTL for grain yield, grain number m^−2^, 1000-grain weight, maturity, plant height, seedling biomass, tiller number, chlorophyll content, leaf symptoms, Na^+^ and K^+^ concentrations of leaves and shoots for a bread wheat mapping population (Berkut/Krichauff). With the recent renewed interest in Cl^−^ [13], we revisited this population to (i) elucidate the genetic control mechanisms of Cl^−^ homeostasis via a QTL approach, and (ii) investigate the relationships among seedling biomass, grain yield and plant Na^+^, K^+^ and Cl^−^ concentrations. Here we report for the first time identification of a major QTL for Cl^−^ concentration in bread wheat and discuss its importance for marker-assisted selection and fine mapping/discovery of genes involved in Cl^−^ transport in bread wheat. We also demonstrate that Cl^−^ accumulation is a polygenic trait, but does not appear to be a reliable predictor of ST based on grain yield alone.

## Materials and Methods

### Plant material

A doubled-haploid (DH) population (152 lines) from a cross between bread wheat (*Triticum aestivum* L.) genotypes Berkut [Irene/Babax//Pastor] and Krichauff [Wariquam//Kloka/Pitic62/3/Warimek/Halberd/4/3Ag3/Aroona] was used in this study. The rationale for screening this population for plant Cl^−^ concentration and subsequent QTL detection was that a previous study revealed significantly lower shoot Cl^−^ accumulation in the Krichauff parent than in the Berkut parent [26].

### Phenotyping and trait analysis

Growth room and field studies were described previously [24], [25]. The data for grain yield, grain number m^−2^, 1000-grain weight, maturity, plant height, seedling biomass, tiller number, chlorophyll content, leaf symptoms and Na^+^ and K^+^ concentrations of penultimate leaves and shoots were reported earlier [24], [25]. In the present study shoot and leaf Cl^−^ concentrations of DH population grown in hydroponics and field trials (Roseworthy, Balaklava and Georgetown in South Australia) characterised by low, moderate and high salinity [24] were determined. Either single (hydroponics, two replicates) or 15–20 plants per entry (field trials, two replicates) were sampled for elemental analysis. Shoot (hydroponics) and leaf samples (field trials) were dried at 65°C for 48 h and dry weights recorded. The dried plant samples were then ground and analysed for Cl^−^, calcium (Ca^2+^) and magnesium (Mg^2+^) concentration using either Inductively Coupled Plasma Optical Emission Spectrometry (ICP-OES) (ARL 3580 B, Appl. Res Lab. SA, Ecublens, Switzerland) [27], [28] or a chloride meter (Model 926, Sherwood, Cambridge, UK). For analysis of Cl^−^ using the ICP-OES method, 0.1 g of ground shoot sample was extracted with hot (95°C) 4% HNO_3_ acid in 50 mL capped polypropylene tubes for 90 minutes, whereas for measurements of Cl^−^ using the chloride meter, 0.5 g of ground sample was digested in 40 mL of 1% HNO_3_ at 85°C for 5 hours in a 54well HotBlock (Environmental Express, Mt. Pleasant, South Carolina, USA). ICP-OES was used initially for analysis of the Cl^−^ concentration of hydroponically-grown plants, but due to prohibitive cost, leaf samples of field-grown plants were analysed using a chloride meter. As two different analytical methods were used for determination of Cl^−^ concentration in plant tissues, a number of samples were analysed using both methods, and the high correlation of the measurements between the two methods (hydroponically-grown samples, r^2^ = 0.98, n = 45; field-grown samples, r^2^ = 0.97, n = 15) indicated that the methods were comparable. As single measurements were taken from each extraction, duplicate analysis (one sample per batch of 24) was carried out to determine the homogeneity of the samples, and relative standard deviations between the two measurements were below 5% in all cases. Chloride concentration was expressed on a dry mass basis (mmol kg^−1^ DW).

### Linkage map and interval construction

Genotyping and construction of a genetic linkage map for the Berkut/Krichauff population was described earlier [24], [25]. The constructed linkage map initially comprised 557 markers across 21 chromosomes. After omission of co-locating markers this was reduced to 403 markers with an average interval distance of 9.16 cM. For computational purposes the alleles of the Berkut (A)/Krichauff (B) population were then converted into 1 and -1 respectively and missing marker scores were imputed using the flanking marker method of Martinez and Curnow [29]. A total of 384 inferred interval markers were then constructed using the mid-point interval method of Verbyla et al. [30].

### Multi-environment analysis

To understand the genetic relationships of the DH lines across the field trials and growth room a multi-environment trials (MET) analysis was conducted for each of the traits Na^+^, K^+^ and Cl^−^. For grain yield the MET analysis was restricted to field sites only. The analysis approach follows Smith et al. [31] which involves a linear mixed model including the parsimonious modelling of genetic effects of the DH lines through an appropriate genotype by environment interaction model and also captures non-genetic sources of variation through the use of separate spatial models for the plot errors at each site. The method initially involves the assessment of the spatial or environmental variation occurring at each site through the investigation of the assumption of variance homogeneity, detection of outliers, and the identification of global trends that may exist across the rows or columns of the experiment. For trends existing due to adjacency of plots in a trial, the model included a separable row by column autoregressive correlation structure. Stronger linear trends in either direction are fitted as fixed effects in the model. Design parameters such as genotypic replication or blocking structures were fitted as separate random effects.

The genotype by environment interaction model involved the use of an unstructured heterogeneous correlation matrix that appropriately captures the genetic relationship of the DH lines between trials. If a strong positive genetic correlation exists between any two trials then the relative performance or rankings of the varieties at each of the trials will be similar. As a consequence these trials will most likely share common or co-locating QTL with a common parent being favoured at each locus. Trials that exhibit little or no genetic correlation between them would exhibit different relative rankings for the varieties and most likely have unshared QTL. Each multi-environment analysis was performed using residual maximum likelihood (REML) and the estimated genetic correlations between sites were extracted for interpretation.

### Multivariate analysis

A multivariate linear mixed model analysis of four traits (Na^+^, K^+^, Cl^−^ and grain yield) was conducted for each field trial in order to estimate genetic correlations of the DH lines between traits. A multivariate analysis was also conducted for the traits in the growth room and included Na^+^, K^+^, Cl^−^, and seedling biomass. For each of the trials, estimates of the genetic relationships between DH lines were modelled through the use of a trait by genotype interaction with an unstructured heterogeneous correlation matrix. The model also included a separable trait by row by column spatial model for the plot errors with an unstructured heterogeneous correlation matrix for the trait component of the model. This unstructured correlation matrix ensures that traits collected from the same trial are connected phenotypically. This separable structure for the spatial model also assumes that the traits have a common row by column separable autoregressive correlation structure. Similar to the MET models, strong trends for any given trait were captured using the appropriate fixed effects, and random effects were used to model genotypic replication as well as blocking structures existing within each trial. Each multivariate analysis was performed using REML and the estimated genetic correlations between traits were extracted for interpretation.

### QTL analysis

For each of the field sites and the growth room the detection and estimation of QTL for the measured traits was accomplished using the R [32] package wgaim [33]. The package is a computational implementation of Verbyla et al. [30], [34]. In this approach, an initial base linear mixed model is established that contains non-genetic effects that account for extraneous variation. These include an appropriate spatial model for the errors as well as fixed and random effects that are relevant to the trial being examined. The base model also includes a random effect term that captures the genetic variation between the DH lines. Following Verbyla et al. [34] the base model is then extended by including the complete set of inferred interval markers into the base linear mixed model as a contiguous block of random effects with a single variance parameter. The significance of this variance parameter is then checked using a simple residual likelihood ratio test. If found significant, an alternative outlier model is formulated and the inferred interval marker with the largest outlier statistic is chosen as a putative QTL. This inferred interval marker is then removed from the contiguous block of random effects and placed as a separate random covariate in the original base model as well as the extended model that includes the remaining set of inferred interval markers. The forward selection process is then repeated until the variance parameter associated with the remaining inferred interval markers is not significant. The complete set of putative QTL selected appears additively as random covariates and is summarised using the methods of Verbyla et al. [34]. The summary includes the left and right flanking markers of the individual QTL, their effect sizes, approximate LOD scores as well as individual contributions to the overall genetic variance.

For all traits analysed in the present study, the best linear unbiased predictions of the genotypes were extracted from the base linear model and were used to calculate a generalized (broad-sense) heritability using the formula developed by Cullis et al. [35].

### Comparative analysis and candidate genes co-located with the 5A Cl^−^ concentration QTL

To align the genetic position of the 5A Cl^−^ QTL to its physical position in the wheat genome, the sequence of RFLP markers with nearby location to the QTL-flanking SSR markers *gwm304* and *barc141* were identified using the database GrainGenes 2.0 (wheat.pw.usda.gov). The sequences of co-located RFLP markers *bcd21* and *psr128* were 451 bp and 430 bp, respectively, and were both derived from ESTs (GrainGenes 2.0). The sequences were used for homology searches using the BLAST tool at https://urgi.versailles.inra.fr/blast/blast.php. The search was carried out against the sequences of all bread wheat chromosomes showing the best hits on 5AL, as expected. The gene sequence hits were used for synteny analysis using the Genome Zipper v5 across wheat, rice, *Brachypodium* and sorghum revealing the syntenic regions between 64 non-rendundant wheat ESTs, *Brachypodium* chromosome 4, rice chromosome 9 and sorghum chromosome 2.

To assign potential functions to the wheat genes underlying the 5A Cl^−^ QTL interval, all 64 non-redundant wheat ESTs were used as queries for homology searches at the National Center for Biotechnology Information (NCBI) using BLASTN against the non-redundant nucleotide database.

## Results

### Responses to salinity stress, distributions and relationships between traits

Data on Cl^−^ concentration provided an opportunity to re-examine the relationships amongst traits associated with ST such Na^+^ and K^+^ accumulation, seedling biomass and grain yield in low (Balaklava), moderate (Roseworthy) and high (Georgetown and growth room) saline environments [24]. Krichauff had 10–20% lower Na^+^ concentration than Berkut in field trials, and these differences diminished in hydroponics, whereas Krichauff had 14–25% lower Cl^−^ concentration than Berkut in all environments ranging from 362–669 and 482–775 mmol kg^−1^ DW for Krichauff and Berkut, respectively (Table 1), similar to previous studies [26]. It was interesting to observe that Cl^−^ concentrations were much higher than Na^+^ concentrations. Berkut had slightly higher K^+^ concentration than Krichauff but only at low to moderately saline field trials at Roseworthy and Balaklava (812–934 and 729–882 mmol kg^−1^ DW for Berkut and Krichauff respectively; Table 1). As for seedling biomass and/or grain yield production under salinity stress, Berkut produced 18% higher seedling biomass than Krichauff in hydroponics, whilst Krichauff had 6–7% higher grain yield in field trials (Table 1). In all traits, there was evidence of transgressive segregation.

**Table 1:** **Parental means, population mean and range for Na^+^, Cl^−^, K^+^, Ca^2+^ and Mg^2+^ concentrations (mmol kg^1^ DW) in penultimate leaves (field trials) and whole shoots (hydroponics), seedling biomass [shoot DW (g plant^1^)] and grain yield (t ha^1^) in Berkut/Krichauff DH population tested for ST in hydroponics and field trials (Roseworthy, Balaklava and Georgetown).**

		Parental Lines		DH population		
Test environment	Trait	Berkut	Krichauff	mean	range	Heritability h^2^
Hydroponics	Na^+^ conc.	298	295	270	194–361	0.58
(100 mM NaCl ∼10 dS m^1^)	Cl^−^ conc.	457	376	378	236–480	0.67
	K^+^ conc.	771	779	749	661–869	0.82
	Shoot DW	1.523	1.296	1.537	1.210–1.835	0.57
	Ca^2+^ conc.	107.2	87.3	97.9	77.4–116.7	0.83
	Mg^2+^ conc.	94.7	77.5	84.1	73.2–95.1	0.77
Roseworthy	Na^+^ conc.	15.2	12.6	12.4	8.8–15.9	0.57
(low salinity, ECe<4 dS m^1^)	Cl^−^ conc.	482	362	393	272–510	0.82
	K^+^ conc.	812	729	739	621–847	0.64
	Grain yield	2.237	2.392	2.116	1.451–2.489	0.76
Balaklava	Na^+^ conc.	13.1	11.6	10.9	7.4–14.5	0.78
(Moderate salinity, ECe = 4–8 dS m^1^)	Cl^−^ conc.	557	447	474	301–598	0.82
	K^+^ conc.	934	882	865	749–1011	0.77
	Grain yield	2.714	2.888	2.671	1.758–3.035	0.81
	Ca^2+^ conc.	121.3	97.2	110.7	71.9–151.4	0.82
	Mg^2+^ conc.	77.2	62.2	73.5	52.0–95.1	0.79
Georgetown	Na^+^ conc.	20.5	16.3	18.7	10.9–30.4	0.60
(High salinity, ECe>8 dS m^1^)	Cl^−^ conc.	775	669	688	571–884	0.84
	K^+^ conc.	1363	1332	1328	1064–1523	0.76
	Grain yield	0.645	0.686	0.573	0.277–0.792	0.72

The means represent predicted values from MET (Na^+^, Cl^−^, K^+^) and single environment (Ca^2+^ and Mg^2+^) analysis of each trait. Broad-sense heritability is also given for individual traits at each environment.

To determine whether selection for Na^+^ and/or Cl^−^ exclusion or even K^+^ accumulation would lead to improved ST, multivariate analysis of concentrations of Na^+^, Cl^−^, K^+^, seedling biomass and grain yield was performed for each of the environments (Table 2). The analysis showed that there was generally a good estimated correlation between Cl^−^ and either Na^+^ or K^+^ with two exceptions: there were negligible correlations for Na^+^ *vs* Cl^−^, and K^+^ *vs* Cl^−^ at Balaklava and in hydroponics respectively. A negative but moderate correlation was observed between seedling biomass and either Na^+^ (r = 0.479) or Cl^−^ (0.527).

**Table 2:** **Estimated genetic correlations between shoot DW (hydroponics), grain yield (field) Na^+^, K^+^, and Cl^−^ (field and hydroponics) extracted from the fitted multi-trait model at each environment.**

Environment	Na^+^ *vs* Cl^−^	K^+^ *vs* Cl^−^	Shoot DW or yield *vs* Na^+^	Shoot DW or yield *vs* K^+^	Shoot DW or yield *vs* Cl^−^
Hydroponics	0.875	0.195	−0.486	−0.146	−0.531
Roseworthy	0.451	0.634	0.200	0.097	0.053
Balaklava	0.122	0.517	−0.200	0.060	0.097
Georgetown	0.319	0.763	0.066	0.148	0.082

To analyse genotype by environment interaction for each of the measured traits, multi-environment analyses and genetic correlations were estimated between environments. Representing low (Balaklava) to moderate (Roseworthy) saline environments, correlations for Na^+^, Ka^+^, Cl^−^ concentrations and grain yield were consistently high between these sites whereas low to moderate correlations were observed for these traits between other higher saline environments (Table 3). Therefore, it is reasonable to assume that selection for similar trait values may not be consistent between higher saline sites, indicating environmental effects controlling ion accumulation and grain yield.

**Table 3:** **Estimated genetic correlations extracted from the fitted multi-environment model for individual traits.**

		Roseworthy	Balaklava	Georgetown
Na^+^ concentration	Balaklava	0.861		
	Georgetown	0.016	0.167	
	Growth room	0.182	0.186	0.464
				
		Roseworthy	Balaklava	Georgetown
K^+^ concentration	Balaklava	0.794		
	Georgetown	0.568	0.598	
	Growth room	0.361	0.322	0.225
				
		Roseworthy	Balaklava	Georgetown
Cl^−^ concentration	Balaklava	0.897		
	Georgetown	0.582	0.462	
	Growth room	0.276	0.427	0.339
				
		Roseworthy	Balaklava	
Grain yield	Balaklava	0.706		
	Georgetown	0.130	0.338	

### Generalized (broad-sense) heritability (*h^2^*)

Estimates of *h^2^* differed with trait and environment, ranging from moderate to high (0.6–0.8) (Table 1). Amongst the mineral elements, Na^+^ concentration had the lowest *h^2^*, while Cl^−^ concentration had the highest *h^2^*. Heritability of shoot DW (0.6) was lower than that of grain yield (0.7–0.8). Heritability of individual traits across environments showed relatively consistent (Ca^2+^, Mg^2+^ and Cl^−^ concentration) to inconsistent patterns (Na^+^ concentration). As reported earlier [24], consistently higher *h^2^* values indicate greater ability for selection for the traits, while lower and variable *h^2^* suggest the presence of substantial environmental effects, difficulty for direct selection, and the need for more replications.

### QTL for Cl^−^ concentration

**Figure 1:**
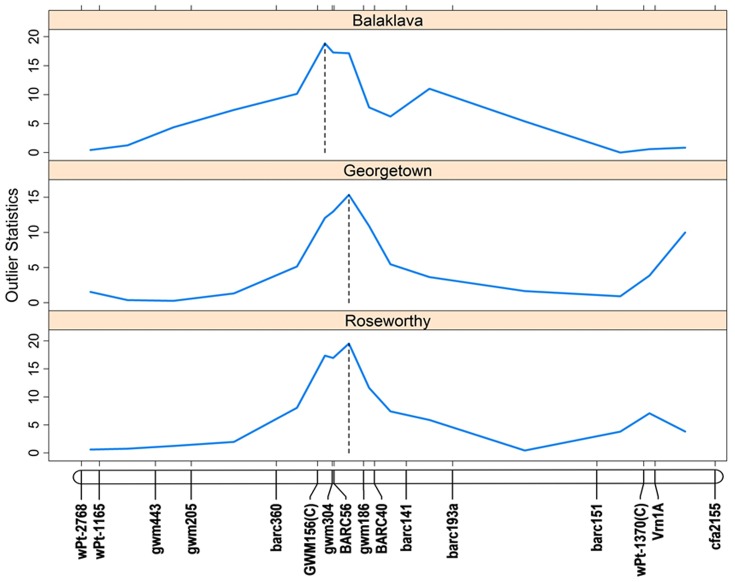
Location of Cl^−^ concentration QTL (*barc56*/*gwm186*) on chromosome 5A detected in field trials (Balaklava, Georgetown and Roseworthy) with varying salinity levels. The outlier statistics represent LOD scores (Table 4).

In the initial analysis in which differences in phenology were not included, there were few Cl^−^ concentration QTL co-locating with QTL for maturity on 5A and 5D (data not shown). Similar to Bonneau et al. [36], after the known differences in the genetic component of the phenology were addressed by fixing the maturity genes in the analyses, 14 QTL were identified (Table 4). However, most QTL were specific to single environments, and only three QTL were detected in two or more environments (3A, 5A, 7D) indicating some genotype by environment interaction. Interestingly, there were no co-located QTL controlling trait variation from hydroponics and field trials. The most significant QTL in hydroponics on chromosome 2A explained 20% of the total genetic variance and the Krichauff allele was responsible for increased Cl^−^ concentration. QTL on chromosome 3A and 7D were detected from multi-location trials and explained 4–11% of the total genetic variance, while QTL on 5A accounted for 27-32% of the total genetic variance (Table 4). Either the Berkut (3A, 5A) or the Krichauff (7D) allele was associated with increased Cl^−^ concentration at these loci. As the two QTL on 5A appear in tandem, these loci were further investigated to determine whether there may just be one rather than two separate QTL. The plot of outlier statistics (Figure 1) shows that there is in fact just one QTL on 5A expressed at all field locations. The QTL detected at one location only accounted for a small proportion of the total genetic variance, varying from 3.6 to 9.9% with either the Berkut or Krichauff allele being associated with increased Cl^−^ concentration (Table 4).

**Table 4:** **QTL associated with Cl^−^ concentration in hydroponics and field trials (Roseworthy, Balaklava and Georgetown) under varying degrees of salinity stress.**

Environment	Ch	Interval	Distance (cM)	Size*	Prob.	% Var.	LOD	**Co-locations with Na^+^ or K^+^ in field locations and hydroponics reported earlier [24], [25]
Roseworthy	2B	*cfd011- gwm374(C)*	88.0–95.8	−12.5	0.001	4.1	2.4	
	3A	*wmc343- cfa2262*	75.7–79.7	20.6	0.000	11.1	6.4	
	3B	*wPt-9049(C)- wPt-0021*	124.4–154.0	13.4	0.002	3.6	2.1	
	5A	*barc56/gwm186*	90.0–100.5	36.8	0.000	32.4	18.3	Na^+^-GT, K^+^-RW, K^+^-BA, K^+^-GT
	6D	*cfd005(C)- gpw95010(C)*	172.8–174.1	−11.7	0.001	3.9	2.4	
	7D	*barc214- gwm437*	130.8–133.5	−11.3	0.002	3.6	2.0	Na^+^-RW, Na^+^- RH, Na^+^-WT
	7D	*wmc014a- wPt-0695*	243.5–246.4	−11.3	0.002	3.6	2.1	
Balaklava	3A	*barc324- wmc343*	67.0–75.7	9.4	0.002	4.4	2.2	
	3B	*gwm247- wPt-4412*	195.1–196.1	13.9	0.000	9.9	4.8	
	5A	*gwm156(C)- gwm304*	84.1–89.3	25.4	0.000	30.8	14.8	
Georgetown	5A	*barc56- gwm186*	90.0–100.5	30.7	0.000	27	11.1	
	7B	*6cg1- wPt-7318*	0.0–22.7	−14.5	0.000	5.4	2.9	
	7D	*barc214- gwm437*	130.8–133.5	13.2	0.001	5.7	2.6	
Hydroponics	1D	*wmc216- wPt-1799*	80.7–131.2	21.3	0.002	5.8	2.0	
	2A	*wPt-3114- wmc170(C)*	92.5–109.9	−33.5	0.000	21.1	7.1	Na^+^- HYDRO, Na^+^-RW, Na^+^-BA
	2B	*wmc272- barc349*	106.5–112.7	−19.4	0.000	8.5	2.7	Na^+^-HYDRO
	2B	*wPt-7859- wPt-7161*	198.9–200.2	18.9	0.000	8.4	2.9	Na^+^-HYDRO
	3A	*barc324- wmc343*	67.0–75.7	17.4	0.001	6.7	2.3	Na^+^-RW
	7A	*gwm282- wPt-0961(C)*	170.2–183.3	−19.3	0.000	7.8	2.7	Na^+^-HYDRO

Only those intervals with *P* values ≤0.01 and LOD>2.0 are presented. Field locations Roseworthy, Balaklava and Georgetown were classified as low, moderate and high salinity, respectively. Please see Genc et al. [24] for soil salinity classification.

*Positive and negative values indicate that Berkut and Krichauff alleles increased the phenotypic values, respectively. **The same co-locations appearing at multiple environments were presented only once. Abbreviations; Hydroponics = HYDRO, Balaklava = BA; Georgetown = GT; Roseworthy = RW. QTL names with letter C indicate several co-locating markers at those loci.

Some QTL for Cl^−^ concentration were co-located with QTL for either Na^+^ or K^+^ concentration from hydroponics and field trials (Table 4). It was interesting to observe that in hydroponics QTL for Cl^−^ concentration were co-located with QTL for Na^+^ concentration (2A, 2B, 2B, 3A, 7A), while in field trials they were co-located with both Na^+^ (3A, 5A, 7D) and K^+^ (5A).

### QTL for calcium (Ca^2+^) and magnesium (Mg^2+^) concentration

As Ca^2+^ and Mg^2+^ nutrition of the plant can also be affected by salinity [26], [37]–[40], their genetic control under salinity stress was also investigated, in a limited way, using a QTL mapping approach. A total of 13 QTL for Ca^2+^ concentration and 6 QTL for Mg^2+^ concentration were identified in hydroponics and the field (Table 5). At those loci, either the Berkut or Krichauff allele increased the concentration of these cations. The most significant QTL for Ca^2+^ concentration was the QTL in hydroponics accounting for 13% of the genetic variance (Table 5). This QTL was also co-located with the Cl^−^ concentration QTL detected in hydroponic conditions (Table 4). For Mg^2+^ concentration, the QTL on 3A in hydroponics was the most significant, explaining 15% of the genetic variance (Table 5). This QTL was also co-located with a QTL for Cl^−^ concentration detected in environments with low to moderately saline conditions (Roseworthy and Balaklava) and hydroponics e (Table 4). However, there were no common QTL that were detected under hydroponics and the field for either of these two cations.

**Table 5:** **QTL associated with Ca^2+^ and Mg^2+^ concentrations in hydroponics and in a field trial (Balaklava) under varying degrees of salinity stress.**

Environment	Trait	Ch	Interval	distance (cM)	Size*	Prob.	% Var.	LOD
Balaklava	Ca^2+^ conc.	3A	*cfa2262-wPt-3816*	79.7–100.95	5.7	0.000	10.6	3.6
		4A	*gwm165a-wmc420*	37.8–49.4	5.1	0.000	9.9	3.2
		5B	*gwm499-wPt-5851*	43.9–46.1	−5.2	0.000	11.4	2.9
		7D	*wmc436b-barc214*	96.8–130.8	4.6	0.001	6.2	2.2
	Mg^2+^ conc.	2A	*gwm294-gdm093(C)*	113.5–150.8	2.4	0.002	6.2	2.1
		4B	*gwm149(C)-wPt-1505(C)*	48.0–48.7	2.3	0.001	9.1	2.3
		5B	*wPt-3457-wPt-1250*	51.5–54.3	−2.4	0.001	10.1	2.2
Hydroponics	Ca^2+^ conc.	1D	*wmc216-wPt-1799*	80.7–131.2	4.9	0.000	13.3	10.1
		2B	*wPt-5707-wPt-3561*	58.4–61.0	−1.9	0.001	3.9	2.5
		3B	*gwm389-wPt-8093(C)*	0.0–0.7	−1.9	0.000	4.0	3.4
		3B	*wPt-4412-wPt-8352(C)*	196.1–197.1	1.6	0.002	2.8	2.1
		4A	*wmc106-gwm165a*	28.4–37.8	2.5	0.000	6.3	4.4
		4D	*gpw95001-gwm165b*	49.7–50.5	−2.9	0.000	9.0	6.5
		5B	*gwm371-gwm499*	37.6–43.9	−2.5	0.000	6.3	3.1
		6B	*cfd076b-wPt-4924*	119.8–124.4	3.4	0.000	11.8	6.6
		7B	*wPt-6372(C)-wPt-3833*	63.2–66.8	2.2	0.000	5.1	3.4
	Mg^2+^ conc.	2A	*gwm294-gdm093(C)*	113.5–150.8	1.6	0.000	6.6	2.8
		3A	*gwm732a-barc1121(C)*	2.2–8.8	−1.4	0.000	7.6	2.9
		3A	*barc324-wmc343*	67.0–75.7	2.1	0.000	15.4	5.5
		4B	*gwm513(C)-gwm495*	43.4–44.7	1.6	0.000	9.9	3.4

Only those intervals with *P* values ≤0.01 and LOD>2.0 are presented. Balaklava location was classified as moderate salinity. Please see Genc et al. [24] for soil salinity classification.

*Positive and negative values indicate that Berkut and Krichauff alleles increased the phenotypic values, respectively. QTL names with letter C indicate several co-locating markers at those loci.

### Known chloride transporters/channel in grass genomes and the 5A Cl^−^ QTL'

In previous studies, employing transcription analysis under salt stress, cellular localization and transgenesis for functional characterization, CLC (chloride channel) and CCC (cation chloride co-transporter) genes had been identified to play a role in Cl^−^ homoeostasis in plants; examples are CLC1 in tobacco and soybean [13], [41], [42], [43] and CCC in Arabidopsis [44].

**Figure 2:**
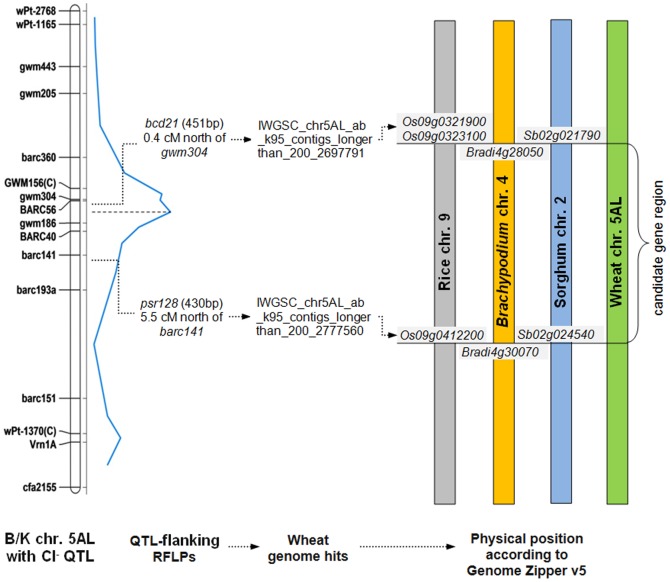
Inferred physical position of the Cl^−^ concentration QTL on 5AL in Berkut/Krichauff identified at Roseworthy field location onto 5AL in wheat.

To investigate the presence of candidate genes such as CLCs, CCCs and other ion transporters within the QTL interval on chromosome 5A, we physically positioned the 5A Cl^−^ concentration QTL in the wheat genome sequence. For this purpose, the gene-based sequences of RFLP markers, *bcd21* and *psr128* with close linkage to the QTL-flanking SSR markers *gwm304* and *barc141* (Figure 2) were used to find wheat genome sequences. As expected, both RFLP sequences had their best hits in bread wheat chromosome 5AL and allowed to retrieve matching contigs of 4.2 kb and 9.1 kb for *bcd21* and *psr128*, respectively. As both RFLPs had originally been derived from ESTs, the corresponding wheat genome contigs (4.2 and 9.1 kb) identified gene hits in rice chromosome 9 (*Os09g0321900* and *Os09g0412200*). These rice genes functioned as borders of the physical interval in the comparative analysis between rice, *Brachypodium* and wheat using the alignment in Genome Zipper v5 (Figure 2). In rice (MSU Release 7 at rice.plantbiology.msu.edu/cgi-bin/gbrowse/rice/#search), the syntenic interval contained 547 genes.

Within the physical interval underlying the 5A Cl^−^ QTL, there were five different classes of genes encoding different transporters or channels: eleven ABC-transporter genes, and single genes encoding a nucleobase-ascorbate transporter, a vacuolar iron transporter (VIT1), a voltage-dependent anion channel (VDAC1) and a potassium transporter (HKT23-like) (Table 6).

**Table 6:** **Candidate genes underlying the physical interval of the Cl^−^ QTL on chromosome 5A.**

*Brachypodium* (v1.2)	Rice (Gene ID at MSU^a^/at IRGSP v2)	Sorghum (v1.4)	Wheat (v5^b^)	Predicted protein function^a^
				
-	-	*Sb02g022750*	-	ABC transporter
*Bradi4g28660*	*LOC_Os09g19734/OS09G0361400*	*Sb02g022910*	*WHE0957_E03_J05ZT; Traes_5AL_99BEC1C3B*	Voltage dependent anion channel 1 (VDAC1)
*Bradi4g29102*	*LOC_Os09g20480/Os09g0371000*	*Sb02g023340*	*Traes_5AL_F8B48EC59*	ABC transporter
*Bradi4g29110*	*LOC_Os09g20490/Os09g0371100*	*Sb02g023370*	-	ABC transporter
*Bradi4g29120*	“	*Sb02g023380*	-	“
*Bradi4g29110*	*LOC_Os09g20500/Os09g0371200*	*Sb02g023370*	-	ABC transporter
*Bradi4g29120*	“	*Sb02g023380*	-	“
*Bradi4g29140*	*LOC_Os09g20510/Os09g0371300*	*Sb02g023360*	-	ABC transporter
*Bradi4g29110*	*LOC_Os09g20520/Os09g0371400*	*Sb02g023370*	-	ABC transporter
*Bradi4g29120*	“	*Sb02g023380*	-	“
*Bradi4g29347*	*LOC_Os09g21000/Os09g0376900*	*Sb02g023620*	*Traes_5AL_B64648FE6*	Potassium transporter family (HKT23-like)
*Bradi4g29440*	*LOC_Os09g21340/Os09g0381100*	*Sb02g023720*	*WHE1104_A05_B10ZS;WHE0807_A06_B11ZS; Traes_5AL_3E0C865DF*	Nucleobase-ascorbate transporter
*Bradi4g29650*	*LOC_Os09g23110/Os09g0394500*	*Sb02g024060*	*Traes_5AL_01A13992D;Traes_5AL_B8B668113*	ABC transporter
*Bradi4g29720*	*LOC_Os09g23300/Os09g0396900*	*Sb02g024130*	*WHE1787_E02_I03ZS; Traes_5AL_F80B422BA*	Vacuolar iron transporter 1 (VIT1)
*Bradi4g29810*	*LOC_Os09g23640/Os09g0401100*	-	*Traes_5AL_678EA44B2*	ABC transporter

a according to MSU Rice Genome Annotation Project release 7, Ensembl Plants release 22 or NCBI;

b Genome Zipper v5.

## Discussion

### Implications of screening for Cl^−^ concentration in hydroponics and field environments

Phenotyping for ST is generally conducted under hydroponic conditions, and results are rarely validated in relevant field environments. Our recent studies in bread wheat demonstrated that phenotyping for Na^+^ exclusion or ST in hydroponics had limited value in predicting field responses, and QTL differed vastly between hydroponics and field locations [24]. Given the renewed interest in Cl^−^ exclusion and ST [13], we analysed Cl^−^ concentration of hydroponically- and field-grown plants of Berkut/Krichauff DH population to determine the value of hydroponics for QTL analysis of Cl^−^ concentration, and elucidate genetic control mechanisms of Cl^−^ accumulation. The results demonstrated that plant Cl^−^ accumulation varied significantly between hydroponics and field trials and as a result, different QTL were identified between the two systems. As was the case with Na^+^ concentration [24], [25], this was most probably due to the two systems being vastly different [24]. These results suggest that there may be very little value in hydroponics testing to predict field responses to Cl^−^ concentration in bread wheat, and future studies should consider field testing or soil-based-pot assays as an alternative.

### ST and Na^+^ or Cl^−^ exclusion

In most studies to date Na^+^ exclusion, and to a limited extent Cl^−^ exclusion, are traits contributing to ST. However, studies that also examined relationships between ST (absolute or relative growth) and Na^+^ or Cl^−^ concentration reported inconsistent correlations [3]–[8], [10], [12], [45]–[48]. It is noteworthy that studies reporting high correlations (r^2^>0.5) analysed either a small number of genotypes or were conducted under controlled environmental conditions [4], [7], [12], [46], [49]. In the present study genetic correlations were investigated using multivariate analysis. Moderate correlations in controlled environments (generally phenotypic) are not uncommon [13], [21]–[23], [25], while such correlations involving a large number of genotypes in the field are almost non-existent [24]. The lack of genetic correlation between Na^+^ and/or Cl^−^ exclusion and grain yield in field studies suggests that other biochemical and physiological processes need to be taken into consideration for identifying mechanisms associated with ST. These results also indicate that a reductionist approach, such as selection for Na^+^ and/or Cl^−^ exclusion only, may not substantially improve ST in bread wheat. A more reliable approach would be to select for grain yield, unless specific physiological traits are shown to have significant and consistent correlations with grain yield under saline environments.

### Ion channels and transporters involved in Na^+^, K^+^ and Cl^−^ homeostasis

Here we aim to introduce a brief discussion on transport of these ions from the soil solution into the root cell and their movements within the plant with respect to ion channels and transporters. However, a greater focus will be placed on Cl^−^, and for Na^+^ and K^+^, readers are referred to recent reviews [41], [50], [51]. It is well established that the initial entry of Na^+^ from the soil solution into the root cell is passive along the concentration gradient [52], and Na^+^ uptake occurs primarily via non-selective cation channels and transporters [41], [51], [53]. However, Na^+^ efflux (from cytosol into vacuole or removal from xylem) as a tolerance mechanism has to be active as it requires energy [41]. Potassium uptake from the soil solution into the root cell is an active process (i.e. moving across the membrane against its concentration gradient) and largely mediated by genes encoding channels and transporters [54]. Chloride uptake can be both active and passive depending on the external concentration. Under non-saline conditions transport of negatively charged Cl^−^ across negatively charged plasma membrane requires energy, therefore is an active process and mediated by transporters, while under saline conditions most Cl^−^ influx across the plasma membrane becomes passive [14]. However, its movement within or out of the plant must be active and aided by transporters [13]. Despite being the most abundant anion in the plant cells, compared to a number of well characterised Na^+^ and K^+^ transporters and encoding genes [41], very little is known about Cl^−^ transport mechanisms and the genes involved. To date two groups of gene families have repeatedly been discussed in relation to Cl^−^ homeostasis: CLCs and CCCs. From limited studies, it appears that CLCs, with their location in endomembranes, are involved in turgor regulation, stomatal movement and NO_3_^−^ transport [55] but not root Cl^−^ uptake [41], while CCCs are involved in long distance transport of Na^+^, K^+^ and Cl^−^ and function as K^+^:Cl^−^, Na^+^:Cl^−^ or Na^+^: K^+^:Cl^−^ co-transporters [44], [56]. However, there may be other gene families involved in Cl^−^ transport, as discussed by Teakle and Tyerman [13].

### Genetic control mechanisms of Na^+^, K^+^ and Cl^−^homeostasis under salinity stress

A good understanding of inheritance of homeostasis of these ions is required for a successful breeding strategy. Our present knowledge of mechanisms of their inheritance is a mere reflection of the number of studies conducted on them. For instance, due to greater focus on Na^+^, we know more about Na^+^ than K^+^ or Cl^−^. The limited studies in rice and wheat suggest that Na^+^ and K^+^ homeostasis under salinity stress are under separate genetic control [25], [57]–[59], although studies in barley [22], [23] found that QTL for Na^+^ co-located with QTL for K^+^, suggesting one or more genes regulating Na^+^ and/or K^+^ transport such as vacuolar sodium-hydrogen antiporter (NHX) genes [22]. As for Cl^−^, to date there have been only four quantitative genetic studies that reported QTL for Cl^−^ concentration; two in rice [20], [21] and two in barley [22], [23]. In those studies as well as in the present study, moderate correlations between Cl^−^ and either Na^+^ or K^+^, and QTL for different ions mapping to the same and/or different regions indicate the presence of common (i.e. CCCs) and specific transporters for the uptake of these ions [i.e. high-affinity potassium (HKT) and (CLCs)] which in turn suggests common and separate genetic control. For instance, in the present study, Cl^−^ QTL on 2A and 5A co-located with QTL for Na^+^ and K^+^ concentration, respectively [24]. A physiological explanation for these co-locations and correlations between cations (Na^+^ and K^+^) and anions (Cl^−^) may be the charge balance between these two groups of ions since the net movement of ions must be balanced so that there is charge equivalence with small difference [13]. Whereas there were no CCC and CLC genes physically close to the Cl^−^ concentration QTL on chromosome 5A there are 15 transporter and channel genes in the physical interval as candidates for the observed Cl^−^ accumulation. Although we have used the latest release of the comparison between wheat ESTs and contigs with sequenced grass genomes (Genome Zipper v5), it is possible that other genes reside in this region in wheat and the microsynteny is less well preserved as it appears to be so far.

Nguyen et al. [22] recently reported a Cl^−^ concentration QTL under salt stress in barley but used an incorrect barley chromosome nomenclature so that the actual chromosome 5H was mislabelled as 7H (other chromosomes were also mislabelled). The physical position of the RFLP markers *ABC324* and *ABC302* that flank their barley Cl^−^ QTL on 5HL [22] suggest a position close to the physical chromosomal region corresponding to the 5AL Cl^−^ concentration QTL reported here. In fact, the physical position of the northern flanking marker *ABC324* (position chromosome 5H: 399,222,591) slightly overlaps with the position of the southern end of the5A QTL flanked by RFLP marker *psr128* (position chromosome 5H: 412,653,548). It is possible that both QTL are caused by orthologous genes in wheat and barley, although this is far from certain as the QTL intervals contain hundreds of genes and the barley QTL was identified under salt stress in a hydroponics system whereas the QTL in wheat was repeatedly observed under salt stressed field conditions but not in hydroponics. Only further work such as fine mapping and gene expression analysis of the candidate genes will prove unequivocally the identity of the underlying gene for the differential Cl^−^ accumulation between Berkut and Krichauff. The present study provides a compelling case that the 5A QTL contains a K^+^:Cl^−^ co-transporter gene several other candidates capable of moving Cl^−^ ions across membranes. However, as this is the first report in wheat, there is clearly a need for testing other mapping populations and genetics resources to identify other Cl^−^ transporter gene(s) to gain a better understanding of genetic control mechanisms of Cl^−^ homoeostasis in crops.

### Genetic control mechanisms of Ca^2+^ and Mg^2+^ accumulation under salinity stress

The inheritance of plant Ca^2+^ and Mg^2+^ accumulation was also investigated, given the reports of salinity-induced nutritional deficiencies such as Ca^2+^ and Mg^2+^ [26], [37], [39], [40] and the importance of maintenance of adequate nutrition for these essential elements to plant growth and yield under salinity stress. To our knowledge, there have only been two studies in barley [22], [23] that investigated inheritance of Ca^2+^ and Mg^2+^ uptake or accumulation under salinity stress. However, only in one study [23] QTL for Ca^2+^ and Mg^2+^ were detected under salinity stress; one QTL for Mg^2+^ concentration on 6H, and three QTL for Ca^2+^ concentration on 1H, 6H and 7H. The QTL on 6H was common not only to Ca^2+^ and Mg^2+^ but also to ST. To our knowledge, this is the first time in the literature that a QTL for a nutrient other than Na^+^ was co-located with ST, providing evidence for the role of Ca^2+^ and Mg^2+^ nutrition in growth and yield under salinity stress. These results also indicate that Ca^2+^ and Mg^2+^ uptake may occur through common as well as independent pathways. In contrast to the barley study, in the present study, none of the Ca^2+^ and Mg^2+^ concentration QTL co-located with each other or ST (measured as seedling biomass or grain yield), suggesting independent genetic control. However, further studies are required to enable better understanding of their genetic control mechanisms.

## Conclusions

As was the case with Na^+^ [24], plant Cl^−^ responses and related QTL differed widely between hydroponics and field tests, indicating substantial genotype and QTL interactions with environments. The results also indicated that hydroponics-based seedling assays may be very limited in their ability to predict field responses to salinity, and soil-based assays may be the second best option after field testing. As Cl^−^ concentration in the plant correlated only moderately with seedling biomass and showed no correlation with grain yield in the field, it does not appear, on its own, to be a reliable physiological parameter to select for in a breeding context, at least in bread wheat. Further research involving other mapping populations/genetic resources is warranted to be definitive. In the short term, selection for grain yield, which is integrative of all tolerance mechanisms, appears a more reliable strategy, while in the long term identification of donors for various physiological traits and subsequently combining them in a genotype (pyramiding) is likely to be the way forward [60]. This latter process can be fast-tracked via marker assisted selection. Finally the presence of several QTL for Cl^−^ concentration indicates that Cl^−^ uptake/accumulation is a polygenic trait. The discovery of a major QTL for Cl^−^ concentration on 5A that co-locates with several candidate genes that could be involved in Cl^−^ transport in bread wheat provides a starting point for further analysis through fine mapping and functional studies.
